# Elevational Gradients Impose Dispersal Limitation on *Streptomyces*

**DOI:** 10.3389/fmicb.2022.856263

**Published:** 2022-05-03

**Authors:** Janani Hariharan, Daniel H. Buckley

**Affiliations:** School of Integrative Plant Science, Cornell University, Ithaca, NY, United States

**Keywords:** biogeography, microbial, bacterial, diversity, soil, assembly, community

## Abstract

Dispersal governs microbial biogeography, but the rates and mechanisms of dispersal remain poorly characterized for most microbial taxa. Dispersal limitation is driven by limits on dissemination and establishment, respectively. Elevation gradients create striking patterns of biogeography because they produce steep environmental gradients at small spatial scales, and these gradients offer a powerful tool to examine mechanisms of dispersal limitation. We focus on *Streptomyces*, a bacterial genus common to soil, by using a taxon-specific phylogenetic marker, the RNA polymerase-encoding *rpoB* gene. By targeting *Streptomyces*, we assess dispersal limitation at finer phylogenetic resolution than is possible using whole community analyses. We characterized *Streptomyces* diversity at local spatial scales (100 to 3,000 m) in two temperate forest sites located in the Adirondacks region of New York State: Woods Lake (<100 m elevation change), and Whiteface Mountain (>1,000 m elevation change). Beta diversity varied considerably at both locations, indicative of dispersal limitation acting at local spatial scales, but beta diversity was significantly higher at Whiteface Mountain. Beta diversity varied across elevation at Whiteface Mountain, being lowest at the mountain’s base. We show that *Streptomyces* taxa exhibit elevational preferences, and these preferences are phylogenetically conserved. These results indicate that habitat preferences influence *Streptomyces* biogeography and suggest that barriers to establishment structure *Streptomyces* communities at higher elevations. These data illustrate that *Streptomyces* biogeography is governed by dispersal limitation resulting from a complex mixture of stochastic and deterministic processes.

## Introduction

For more than a century, elevational gradients have yielded unique insights into the ecological and evolutionary mechanisms that generate patterns of biogeography (Grinnell, 1917). Steep elevation gradients generate rapid shifts in habitat characteristics over short spatial distances, a property that is useful in determining the degree to which dispersal is driven by spatial distance or variation in habitat characteristics (Sundqvist et al., 2013). Elevation gradients have a strong influence on the biodiversity of plants and animals (Peters et al., 2016) with many taxa exhibiting mid-elevation peaks or “hump-shaped curves” in alpha diversity (Rahbek, 2004; Moradi et al., 2020), and similar patterns have been observed for microbes (Fierer et al., 2011; Singh et al., 2012; Liu et al., 2016; Siles and Margesin, 2016). Elevation can affect biodiversity by a range of mechanisms including ecological filtering by habitat preference (Fierer et al., 2011; Wang et al., 2012; Shen et al., 2014; Cho et al., 2018), variation in carrying capacity, and historical processes linked to patterns of climate change (Badgley and Fox, 2000; Rickart, 2001; de la Giroday et al., 2011; Schai-Braun et al., 2020). In addition, taxa that occupy mountain habitats are uniquely affected by historical climate change as warming climates tend to push species distributions toward higher elevations (Flesch, 2019; Marshall et al., 2020; Neate-Clegg et al., 2021), minimizing dispersal opportunities for species of plants and animals found at high elevations (Sekercioglu et al., 2008).

Biogeographical patterns can be driven by ecological mechanisms (*e.g.*, assembly processes driven by ecological filtering and ecological drift), evolutionary mechanisms (*e.g.*, speciation due to selection and drift), and historical contingency (*e.g.*, neutral processes linked to variation in geology and climate over time) (Hanson et al., 2012). Microbial biogeography is often thought to be constrained by ecological filtering, under the assumption that dispersal is largely unlimited and environmental gradients impose spatial structure on communities due to selection (Navarrete et al., 2015; Liu et al., 2018; Malard and Pearce, 2018; Malard et al., 2019). However, most evidence for unlimited microbial dispersal is obtained using highly conserved taxonomic markers (*e.g.*, rRNA genes) that have low taxonomic resolution and are insensitive to evolutionary processes that drive diversification (Hanson et al., 2012). Studies that use higher resolution taxonomic markers often find evidence for dispersal limitation with evidence that neutral processes can play a role in shaping patterns of microbial biogeography (Whitaker et al., 2003; Polz et al., 2013; Andam et al., 2016b; Choudoir et al., 2016; Choudoir and Buckley, 2018).

To explain the mechanisms that give rise to microbial biogeography we must first understand the forces that govern microbial dispersal. Dispersal is a two-part process comprised of dissemination, the movement from one place to another, and establishment, the successful colonization of a site characterized by the ongoing production of viable offspring (Martiny et al., 2006). Dissemination can be either passive (as driven by wind, erosion, currents, and organismal vectors) or active (as driven by motility or hyphal growth) (Yang and van Elsas, 2018). It is likely that capability for dissemination varies considerably between microbial taxa. For example, windborne dissemination is likely to vary in relation to cell size (Wilkinson et al., 2012), and cell shape likely influences microbial dissemination and establishment (Young, 2006). In addition, dissemination is influenced by environmental states. For example, soil texture and temperature influence spore transport in *Phytophthora* fungi (MacDonald and Duniway, 1978), and weather patterns can affect aerial dissemination (de Groot et al., 2021). Successful dissemination, however, is insufficient for successful dispersal, as microbes must still establish a sustainable population in the new site. Establishment requires that the habitat be suitable for growth, and that competitive interactions (*e.g.*, antagonism, or density-dependent blocking) do not prevent ongoing reproduction (Woody et al., 2007; Cheong et al., 2021).

We performed analysis of *rpoB* amplicons to investigate community assembly in *Streptomyces* along an elevational and spatial gradient in the Adirondacks region in New York State. The use of *rpoB* as a taxonomic marker for this genus improves taxonomic resolution significantly (Rong and Huang, 2012; Higgins et al., 2021) as compared to analyses made using 16S rRNA genes. The use of high-resolution taxonomic markers is essential for investigating the mechanisms that govern microbial biogeography (Hanson et al., 2012; Chase et al., 2017).

*Streptomyces* are bacteria that form aerial hyphae and arthrospores (Flärdh, 2003), which facilitate dissemination. They are common in soil habitats worldwide where they degrade a variety of common substrates derived from plant biomass (Yeager et al., 2017) and produce diverse antibiotics and secondary metabolites (Watve et al., 2001). Despite their high capacity for dissemination, and broad habitat tolerance, *Streptomyces* have been shown to exhibit endemism at regional scales, indicative of dispersal limitation (Andam et al., 2016a). Their wide distribution, ecological significance, theoretical capability for high dispersal, and their observed limited ranges make the *Streptomyces* genus an ideal group to understand dispersal and biogeography patterns in the soil.

We hypothesized that dispersal limitation would also occur at local scales, with dispersal limitation driven by barriers to establishment (*i.e.*, ecological filtering) rather than barriers to dissemination. To evaluate this hypothesis, we examined *Streptomyces* communities at two locations in the Adirondacks region of New York State. Sites at Whiteface Mountain varied greatly in elevation, while sites at Woods Lake varied little in elevation. All sites were broadly similar in habitat characteristics other than those linked to elevation. We predicted that high rates of local dissemination, coupled with ecological filtering as driven by elevation, would produce a strong gradient of beta diversity at Whiteface Mountain, and little variation in beta diversity at Woods Lake. We also predicted that ecological filtering would cause *Streptomyces* taxa to exhibit phylogenetic conservation with respect to elevation preference.

## Materials and Methods

### Soil Sampling for Whiteface Mountain and Woods Lake

**FIGURE 1:**
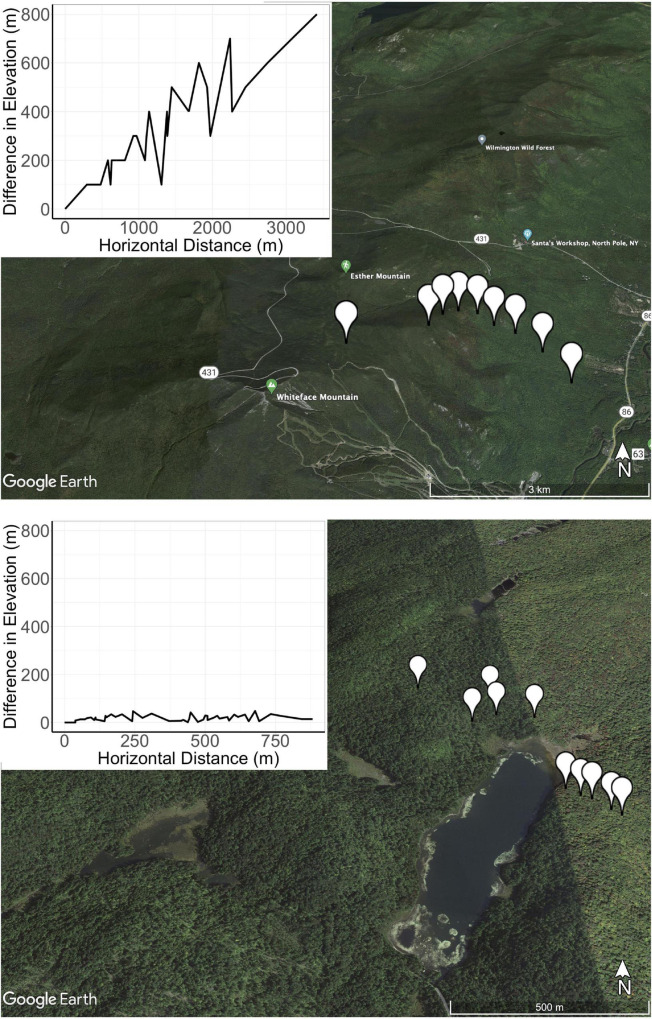
The sites at Whiteface Mountain (WM; **top**) span more than 1,000 m elevation while those at Woods Lake (**bottom**) span less than 100 m elevation. The topographical profile is provided in the inset. Horizontal distances (X-axis) are measured as geodesic distance or the shortest distance between the GPS coordinates of each site while the Y-axis represents difference in elevation relevant to the base elevation at WM.

Soil samples were collected from nine locations on Whiteface Mountain (WM) in New York State (Figure 1). The average elevation change and horizontal distance between sites at WM was 347 and 1,361 m, respectively (metadata described in Table 1). The top of the mountain consists of shallow and well-drained loamy soil (Lythic Cryofolist) with moderately deep, well-drained Wallface-Skylight soils on gneiss bedrock at 1,200 m. The soil type changes to frigid Lithic Haplohumods characteristic of glaciated uplands below 800 m, and the base of Whiteface Mountain consists of deep, excessively drained sandy loam (Typic Haplorthod). To contrast the elevational gradient found at WM with the effects of spatial distance, we also collected samples from ten locations spanning two watersheds of Woods Lake (WL), which is situated in the Adirondacks region, 190 km from WM (Figure 1). Details of soil collection from Woods Lake are described elsewhere (Melvin et al., 2013). The average elevation change and horizontal distance between sites at WL was 19 and 353 m, respectively. WL contains sandy glacial till soils on top of hornblende granitic gneiss bedrock (Orthod Spodosols). Only non-limed soils from the Woods Lake watershed were included in this study.

**TABLE 1:** Spatial and environmental metadata for each sample at Whiteface Mountain and Woods Lake.

Sample	Elevation (m)	pH	Temperature	SOM	Watershed
S01_R01	1,200	3.93	15	16.6288192	NA
S01_R02	1,200	4.1	15	31.9330525	NA
S01_R03	1,200	3.74	15	29.1874668	NA
S02_R01	1,100	3.86	16	22.1387585	NA
S02_R02	1,100	3.61	17	53.9605634	NA
S02_R03	1,100	3.86	16	35.9873995	NA
S03_R02	1,000	3.67	16	26.3294986	NA
S03_R03	1,000	4.05	17	27.4130845	NA
S04_R02	900	3.93	17	33.735336	NA
S04_R03	900	4.69	17	27.6732296	NA
S05_R01	800	4.42	19	15.7430731	NA
S05_R02	800	4.52	19	14.0397492	NA
S05_R03	800	4.45	18	14.61	NA
S06_R01	700	4.96	20	17.9205852	NA
S06_R02	700	4.54	19	22.4786858	NA
S06_R03	700	4.95	18	13.2271829	NA
S07_R02	600	5.09	20	7.89907312	NA
S07_R03	600	4.7	19	8.87326813	NA
S08_R01	500	5.08	20	5.65392979	NA
S08_R02	500	4.81	20	5.04150015	NA
S08_R03	500	4.95	20	6.75924035	NA
S09_R01	400	4.81	19	11.4845938	NA
S09_R02	400	5.07	19	28.7365177	NA
S09_R03	400	5.11	19	9.08149529	NA
C1A1	610	4.14	NA	NA	C1
C1B1	615	4.1	NA	NA	C1
C1C1	657	4.28	NA	NA	C1
C1D1	620	4.16	NA	NA	C1
C1E1	638	4.59	NA	NA	C1
C2A1	609	4.45	NA	NA	C2
C2B1	622	4.62	NA	NA	C2
C2C1	630	4.28	NA	NA	C2
C2D1	643	4.21	NA	NA	C2
C2E1	643	4.88	NA	NA	C2

*Sites that start with C (e.g., C1A1) are in WL, and sites that start with S (e.g., S01_R01) are in WM.*

At each sampling site, soil cores were collected in triplicate using a soil probe (2.5 cm diameter, 5 cm depth). Soil temperature was measured at the time of sampling. Samples used to test soil properties were air dried for 24 h and then sieved using a 2 mm mesh to remove plant debris and rocks, while soil used for DNA extraction was continuously stored at −20°C. Soil pH was measured using the 1:1 soil:water method described elsewhere (Kalra, 1995), and soil organic matter (SOM) content was measured by the loss-on-ignition method described in the Kellogg Soil Survey Laboratory Methods Manual (Burt, 2014).

### DNA Extraction and Sequencing

DNA was extracted using the MoBio PowerSoil^®^ DNA Isolation kit (Qiagen, Germantown, MD, United States) and quantified using the PicoGreen fluorometric assay (Thermo Fisher Scientific, Waltham, MA, United States). A 406 bp region of the RNA polymerase gene (*rpoB*) was amplified by PCR (∼25 ng DNA in a 25 μl reaction) using *Streptomyces*-specific primers Smyces_rpoB1563F and Smyces_rpoB1968R as described elsewhere (Higgins et al., 2021). The PCR reactions consisted of 25 ng DNA, 12.5 μl of Q5 Hot Start High-Fidelity 2X Master Mix (New England Biolabs, Ipswich, MA, United States), 0.625 μl of 4X Quant-iT PicoGreen dsDNA assay reagent (Thermo Fisher Scientific, Waltham, MA, United States), and 1.25 μl each of 10 μM dual-barcoded forward and reverse primers modified for Illumina sequencing as described in Kozich et al. (2013). PCR products from triplicate reactions were pooled and normalized using the SequelPrep Normalization Plate Kit (Thermo Fisher Scientific, Waltham, MA, United States). Fragments of 450 bp in length were size selected with a 1% agarose gel and subsequently extracted and purified from the gel band. Pooled samples were concentrated to 2 ng/μl using a vacuum concentrator and sequenced on an Illumina MiSeq instrument (2 × 300 bp) at the Biotechnology Resource Center, Cornell University.

### Additional Datasets

In addition to the data generated from WM and WL, we also looked for evidence of elevational gradients in a larger dataset generated from soil samples obtained as part of the North American Soil Geochemical Landscapes Project (United States Geological Survey [USGS], 2012). This dataset consists of *Streptomyces rpoB* amplicons generated from 1,108 soil samples derived from sites across the United States and Mexico, spanning 7–3,483 m in elevation (average elevation 674 m). These *rpoB* amplicons were generated using the same primers and protocols described above (Steven Higgins, unpublished).

### Data Analysis

Paired-end reads were joined using bbmerge (Bushnell et al., 2017) and trimmed using Trimmomatic-0.38 (Bolger et al., 2014). Sequences were dereplicated and size-sorted prior to OTU clustering at 99% identity using USEARCH (Edgar, 2010). The 0.99 similarity threshold corresponds to the species cut-off for *Streptomyces*, and provides better resolution for classifying *Streptomyces* at the species level than the 16S rRNA gene (Rong and Huang, 2012; Andam et al., 2016b; Higgins et al., 2021). OTUs were classified with SINTAX (Edgar, 2016). Sequences were aligned using MAFFT v7.475 (Katoh and Standley, 2013) and phylogenetic trees were constructed using the maximum likelihood method with RAxML 8.2.12 (Stamatakis, 2014).

Samples that had fewer than 18 sequences (first quartile value) were discarded from further analyses. All other samples were normalized using the Cumulative Sum Scaling method (Paulson et al., 2013), wherein OTU relative abundances within each sample are divided by the sample’s library size (total number of reads in the sample). Downstream analyses for beta diversity estimates, phylogenetic signal, and phylogenetic clustering were performed using the phyloseq and picante R packages (Kembel et al., 2010; McMurdie and Holmes, 2013). Distance-decay relationships were quantified using the mgram function in the ecodist package (Goslee and Urban, 2007). The relative contributions of ecological processes like drift, selection and dispersal to community assembly were assessed using methods and code described elsewhere (Anderson et al., 2011; Stegen et al., 2013). The indicspecies R package (Cáceres and Legendre, 2009) was used for indicator species analyses, and RAxML (Stamatakis, 2014) was used to reconstruct phylogenetic relationships. Phylogenetic trees were visualized using iTol (Letunic and Bork, 2021). All analyses were performed in R version 3.6.1.

## Results

### *Streptomyces* Diversity at Whiteface Mountain and Woods Lake

**FIGURE 2:**
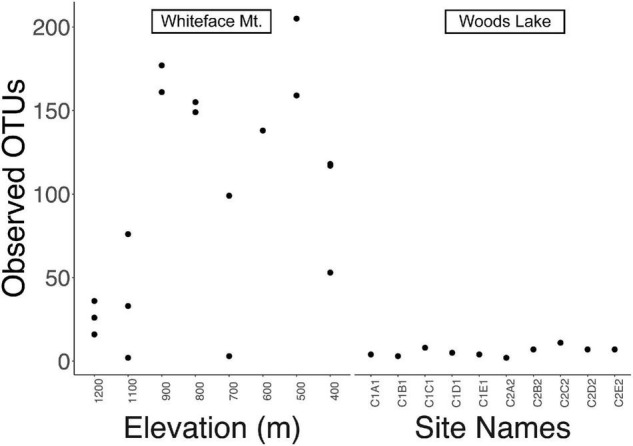
Alpha diversity is represented as number of observed OTUs in each sample. *Streptomyces* at Whiteface Mountain (**left**) exhibited maximal richness at mid elevations with decreasing alpha diversity at the highest and lowest sites. *Streptomyces* at Woods Lake (WL; **right**) exhibited little change in alpha diversity with respect to elevation change. Sample names are provided instead of elevations in the right panel because elevation does not vary across the WL watershed (largest elevation difference is 33 m, Table 1).

*Streptomyces* exhibited greater richness at WM (74 ± 69 OTUs, average and standard deviation) than at WL (6 ± 3, ave. and s.d.), and this result was significant (Mann Whitney *U*-test, *p* = 0.004). *Streptomyces* richness at WM was maximal at mid elevations (500–900 m, Figure 2), consistent with the classic hump-backed pattern of alpha diversity seen in previous studies (Singh et al., 2012; Liu et al., 2016; Kou et al., 2021). In contrast, sites at WL varied little in richness (Figure 2). Although WL had fewer *Streptomyces* OTUs than WM, rarefaction curves indicate that both sites were adequately sampled (Supplementary Figure 1).

*Streptomyces* exhibited greater beta diversity at WM (0.76 ± 0.28, unweighted UniFrac distance, ave. and s.d.) than at WL (0.35 ± 0.21, unweighted UniFrac distance, ave. and s.d.), and this difference was significant (Mann Whitney *U*-test, *p* < 0.0001). In addition, *Streptomyces* communities at WM and WL were highly dissimilar (0.84 ± 0.164, unweighted UniFrac distance, ave. and s.d.). Analysis of similarities (ANOSIM) indicates that elevation is the strongest predictor of beta diversity at WM (*R* = 0.5284, *p* < 0.001, 9,999 permutations). Additionally, beta diversity was partitioned into turnover and nestedness (Baselga, 2010). Species turnover underlies most of the beta diversity at both sites (90.91% at WM, 59.5% at WL), and turnover was significantly higher than nestedness at WM (paired *t*-test, *p* < 0.0001; Supplementary Table 1). Taken together, these results indicate that dispersal is limited between WM and WL, and it is limited across elevation at WM.

A high amount of species turnover indicates that species are replaced from local pools rather than from the regional meta-community. Only 12 OTUs were shared between WM and WL. Ten of these shared OTUs have high relative abundance as compared to random expectations based on a random draw from the regional meta-community (paired *t*-test, 1000 permutations, *p* = 0.002), indicating that high-abundance OTUs are more likely to be shared at regional scales than we would expect due to chance.

### Elevation Drives Community Structure on Whiteface Mountain

**FIGURE 3:**
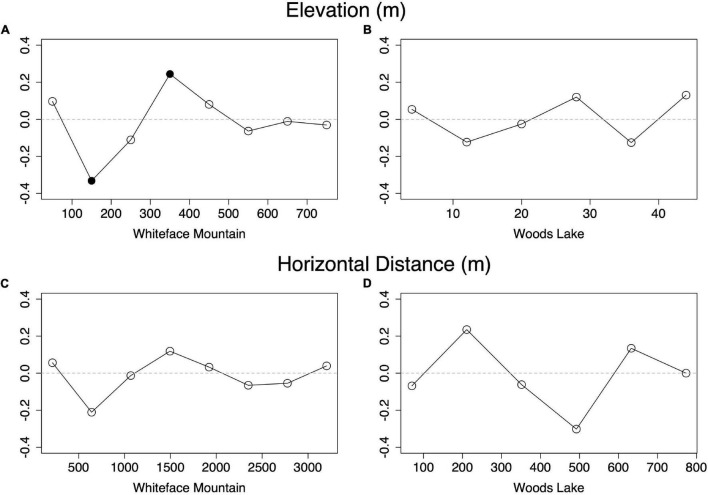
Partial Mantel tests indicate that beta diversity varies significantly with respect to elevation at Whiteface Mountain **(A)** but not at Woods Lake **(B)**, and that beta diversity does not significantly correlate with horizontal distance at either site **(C,D)**. Filled points indicate significant correlation (*p* < 0.01).

If elevation causes phylogenetic clustering, beta diversity should correlate with elevation change. Of the five variables tested, elevation was the only variable that was significantly correlated with beta diversity (Figure 3 and Supplementary Figure 2). The correlation coefficients indicate an intermediate-strength relationship, consistent with previous findings of dispersal limitation in other bacterial communities (Bell, 2010; Angermeyer et al., 2016). Beta diversity at WL was not significantly correlated with elevation, horizontal distance, pH, or any other measured variables.

**FIGURE 4:**
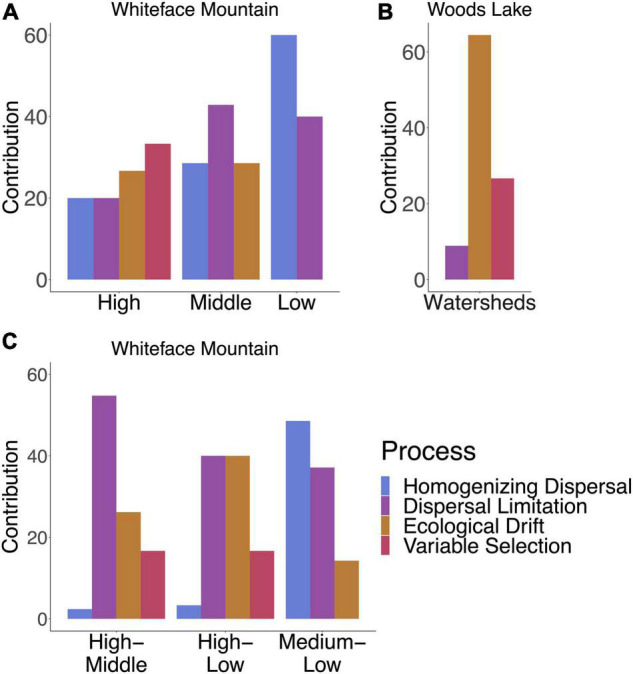
Relative contributions of selection, dispersal, and ecological drift to community assembly vary between sites and within elevation zones in Whiteface Mountain. For WM, High indicates elevations above 1,000 m, Low indicates elevations below 500 m, and Middle indicates 500–1,000 m. **(A)** Variable selection drives beta diversity at sites above 1,000 m in WM while lower elevations have higher levels of homogenizing dispersal resulting in reduced beta diversity. **(B)** Dispersal limitation is highest between sites above 1,000 m and the rest of the mountain, while dispersal plays an important role in homogenizing *Streptomyces* communities between lower elevations. **(C)** Community assembly at WL is dominated by ecological drift with some variable selection.

Community assembly was subdivided into selection, dispersal, and ecological drift [as described by Stegen et al. (2012, 2013)]. Ecological drift was the dominant assembly process within WL. At WM, variable selection was the dominant assembly process at high elevation (above 1,000 m), as expected if ecological filtering is driven by elevation (Figure 4A). However, at WM, the importance of homogenizing dispersal increases at elevations below 1,000 m (Figure 4A). This result suggests that dissemination at WM is driven by downward movement of soil and water from high to low elevation, with ecological filtering due to variable selection limiting the establishment of “high elevation clades” at mid and low elevation sites, while homogenizing dispersal is more common between mid and low elevation sites (Figure 4B).

### Indicator Species Analysis

**FIGURE 5:**
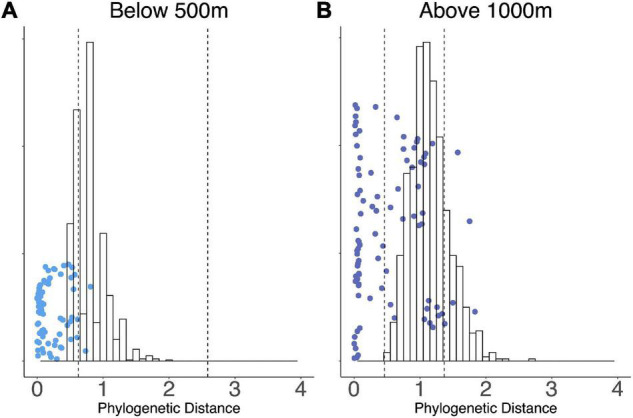
Scatter plots show the distribution of pairwise phylogenetic distances **(A)** between indicator OTUs below 500 m, and **(B)** between indicator OTUs above 1,000 m. The histograms represent a bootstrapped distribution of 1,000 random draws from pairwise phylogenetic distances within the WM community, and 95% confidence intervals are indicated by vertical dashed lines. Each dot represents the phylogenetic distance between a pair of OTUs in that respective category.

We conducted indicator species analysis to identify OTUs specific to elevation zones in WM (multipatt function, indicspecies R package). 16 OTUs were associated with elevations above 1,000 m and 14 OTUs with elevations below 500 m. We evaluated pairwise phylogenetic distance of the indicator species with respect to elevation (Figure 5). Low-elevation indicator species exhibited more phylogenetically similarity to each other than expected due to chance (*t*-test, *p* = 0.006), while high-elevation indicator OTUs were more diverse indicating the presence of multiple phylogenetic clusters (Figure 5).

### Evaluating Indicator OTU Distribution in a Continental-Scale Dataset

**FIGURE 6:**
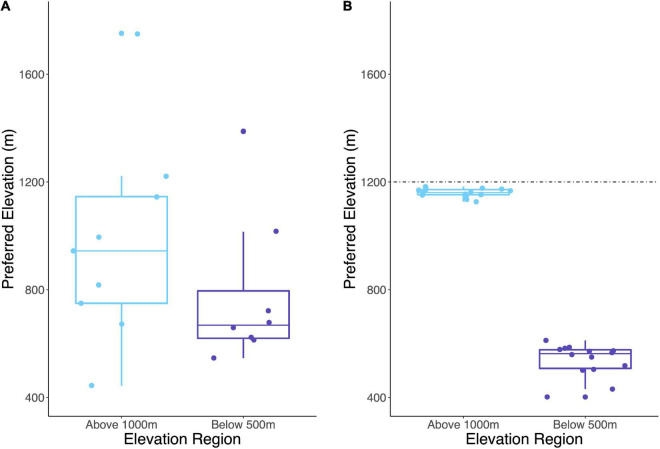
Abundance-weighted average elevation of each indicator OTU or its conspecific representative is represented as preferred elevation for **(A)** continental-scale distribution of *Streptomyces* and **(B)** the distribution of *Streptomyces* at WM. The dashed line indicates maximum elevation sampled at WM. Preferred elevations are significantly different between High and Low indicator OTUs at WM (Welch’s t-test, *p* < 0.001).

We further evaluated the elevational preferences of indicator OTUs by examining their distribution in a continental-scale dataset of *Streptomyces* biogeography. The continental-scale dataset contains *rpoB* sequences from *Streptomyces* communities across North America (see “Materials and Methods” section). Briefly, indicator OTUs from WM were identified in the continental-scale dataset by clustering at 99% identity. The preferred elevation for these OTUs was calculated as the normalized abundance-weighted average of elevations for all sites at which the OTU was detected. Elevational preference at WM had a strong influence on elevational distribution for conspecific OTUs in the continental survey (Cohen’s *d* = 0.7, Figure 6).

**FIGURE 7:**
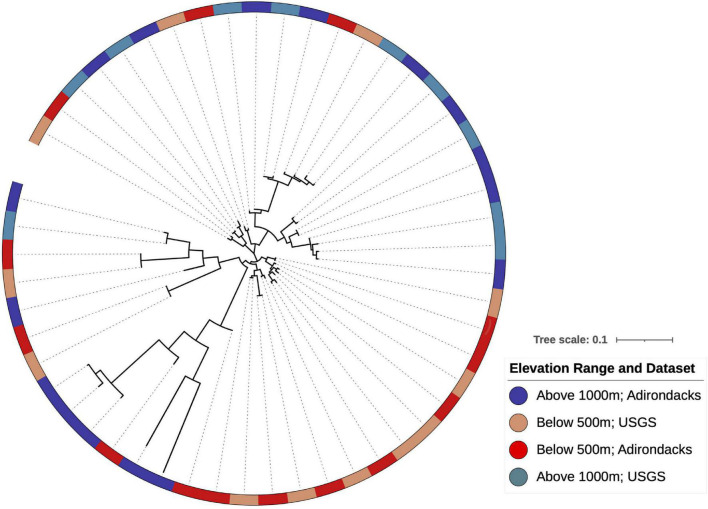
Phylogenetic reconstruction of indicator OTU lineages shows clade-level conservation of elevational preferences within *Streptomyces* found at WM. Some clades show mixed elevation preferences with branch lengths suggesting that indicator OTUs above 1,000 m have greater evolutionary divergence relative to those found at lower elevations. Colors are indicative of elevation and dataset for each OTU as indicated in the legend.

Phylogenetic reconstruction of WM indicator OTUs and their continental relatives (Figure 7) indicated that mixed clades (clades with both high- and low-indicator OTUs) were more evolutionarily divergent than clades with only one indicator type as quantified by mean pairwise phylogenetic distances within each clade (*t*-test with 1,000 permutations, Bonferroni corrected *p* = 0.002). Additionally, clades with only high-elevation OTUs contained less divergence than those with only low-elevation OTUs (*t*-test with 1,000 permutations, Bonferroni corrected *p* = 0.006).

Patristic distance calculations suggest that low-elevation clades in Figure 7 are younger than the mixed or high-elevation clades, as they have shorter root-to-tip distances (0.09 ± 0.06 as compared to 0.16 ± 0.11 for high-elevation OTUs; Kruskal-Wallis test, *p* = 0.004). Hence, OTUs that are now localized above 1,000 m exhibit greater evolutionary divergence than those present at lower elevations.

## Discussion

We show that *Streptomyces* communities are dispersal limited at local spatial scales and that dispersal limitation is governed by limits to both dissemination and establishment. *Streptomyces* communities at Woods Lake had low rates of dispersal, resulting in ecological drift at spatial scales spanning hundreds of meters. Low dispersal could result from low rates of dissemination, but the ability of *Streptomyces* to make aerial hyphae and desiccation resistant spores, and the fact that long range dispersal has been observed (Andam et al., 2016b; Choudoir et al., 2016; Higgins et al., 2021), suggest that limits to dissemination are unlikely to structure communities at local scales. Given the minimal habitat variation among sites at WL, and the broad habitat suitability expected for *Streptomyces*, it also seems unlikely that low dispersal at WL is driven by ecological filtering. Hence, we hypothesize that low dispersal and ecological drift at WL is likely driven by biotic interactions such as antagonism or density dependent blocking (Waters et al., 2013). *Streptomyces* are well known to produce diverse antimicrobial compounds, and other secondary metabolites, that alter biotic interactions (Kinkel et al., 2014; Schlatter and Kinkel, 2014; Vaz Jauri and Kinkel, 2014; Essarioui et al., 2016; Otto-Hanson and Kinkel, 2020). It seems likely that biotic interactions generate barriers to establishment that govern the structure of *Streptomyces* communities at WL.

We see strong evidence of *Streptomyces* dispersal limitation at Whiteface Mountain, with elevation having significant impacts on both dissemination and establishment. Certain clades preferentially occupy either high or low elevation habitats, indicating that barriers to establishment alter community structure across the mountain. The fact that these elevational habitat preferences are also observed in a continental-scale dataset suggests that ecological filtering by habitat preference constrains *Streptomyces* community structure across elevation. However, we also observe that homogenizing dispersal increases toward the base of the mountain (Figure 4), and this suggests that the elevation gradient favors dissemination, likely due to movement of material down the mountain. Both alpha and beta diversity are significantly higher at WM than WL, consistent with the expectation that strong environmental gradients amplify patterns of microbial diversity despite high rates of dissemination. We expect that competitive interactions influence *Streptomyces* community composition at both WL and WM, but that the effect of elevation on dissemination and establishment is the main driver of community structure at WM.

Previous studies offer conflicting evidence for the effect of elevation on microbial biogeography. While research across a montane elevational gradient in Peru showed no effect of elevation on bacterial communities in soil (Fierer et al., 2011), a similar analysis on soils from the Andes Mountains found that bacterial and fungal diversity both varied with respect to elevation (Nottingham et al., 2018). Other studies have documented variation in bacterial community structure across elevation, attributing such variation to a range of factors including soil pH (Cho et al., 2018), aspect (Wu et al., 2017), soil carbon (King et al., 2008), and seasonality (Lazzaro et al., 2015; Zhu et al., 2020).

Conflicting evidence on the relationship between bacterial biogeography and elevation could result from variation in spatial scales, habitat variability, and the phylogenetic resolution of taxonomic markers. Several environmental variables can co-vary with elevation (Sundqvist et al., 2013), making it difficult to disentangle the effect of elevation as opposed to other co-varying gradients. We also know that the spatial scale and taxonomic resolution at which diversity is measured can influence our ability to observe patterns of biogeography (Bent et al., 2003; Martiny et al., 2011; van de Guchte, 2017). Most prior studies of microbial diversity across elevation gradients have been performed using the 16S rRNA gene as a taxonomic marker. The low phylogenetic resolution of this marker makes it unsuitable for assessing mechanisms of dispersal limitation (Choudoir et al., 2012). For example, common taxonomic units defined on the basis of the 16S rRNA gene encompass strains whose ancestors may have diverged 50–150 million years ago (Ochman et al., 1999), and such taxonomic units lack the resolution needed to resolve the mechanisms that underlie extant patterns of microbial biogeography (Hanson et al., 2012). Our ability to identify the effect of environmental gradients on species distributions improves in proportion to the phylogenetic resolution at which diversity is characterized (Ramirez et al., 2018). Many phenotypic traits are conserved among closely related strains (Martiny et al., 2015; Barnett et al., 2021), and so experiments that use taxon-specific, fine-scale phylogenetic markers are vital to illustrate the processes driving microbial biogeography. Several examples of non-16S gene markers already exist in the literature; *dsrA*, *nirK*, *nirS*, and other MLST-based schemes have been used to detect biogeographical patterns in environmental bacteria (Whitaker et al., 2003; Boucher et al., 2011; Angermeyer et al., 2016; Kou et al., 2021; Liao et al., 2021). In this study, the use of a *Streptomyces*-specific amplicon marker allows the exploration of phylogenetic patterns at a fine scale, sufficient for exploring the mechanisms that govern microbial dispersal.

Contemporary and historical climate variation is likely to influence patterns of *Streptomyces* biogeography. Elevation has a clear impact on temperature, as land temperatures decline 0.42°C for every 100 m of elevation, such that a 200 m change in elevation approximates the temperature shift associated with a 1° change in latitude (Montgomery, 2006). Phylogenetic conservation of thermal traits has been shown to influence *Streptomyces* dispersal across latitude (Choudoir and Buckley, 2018), and such thermal adaptation likely contributes to the latitudinal diversity gradient observed for North American *Streptomyces* (Andam et al., 2016b). Whiteface Mountain is one of the highest peaks in the Adirondacks (1,484 m above sea level). Geological evidence indicates that the mountain was glaciated along with the entire Adirondacks region, until glacial retreat about 10,000 years ago (Franzi et al., 2000). This geological timeline means that *Streptomyces* have arrived fairly recently to WM and WL and hence the time for local diversification was limited. Prior to the period of glacial retreat, about 12,000 years ago, the climate in the Adirondacks region would have been approximately 2°C cooler than current conditions (Kaufman et al., 2020). Over time, as the climate warmed, species adapted to warmer climates would have dispersed into the wider Adirondacks region while cold adapted species would have found their habitat restricted to higher and higher positions on the mountain. In this scenario, we hypothesize that ecosystem properties linked to climate variation influence microbial dispersal in soils by controlling the probability that species are able to establish at new sites. Changes in elevation influence a wide range of ecological variables both above and belowground (Sundqvist et al., 2013), and so it would be imprudent to conclude that temperature is the most important variable delimiting establishment, but it seems fair to conclude that ecological properties associated with climate variation can be expected to alter patterns of microbial establishment in soils.

Our findings indicate that a mixture of stochastic and deterministic processes govern *Streptomyces* dispersal. *Streptomyces* form aerial hyphae that produce desiccation-resistant, hydrophobic spores and their physiological traits should support broad habitat tolerance. As a result, we would generally assume that *Streptomyces* have a greater dispersal capacity than most other soil bacteria. However, we found high dissimilarity in communities that occupied similar habitats and similar elevations (350–450 m elevation) at both WL and WM. This result suggests local limits on dispersal, likely driven by capacity for establishment as determined by competitive interactions between existing species and new immigrants. However, we did see evidence for homogenizing dispersal at the base of Whiteface Mountain suggesting that high rates of dissemination, likely driven by mass transport down the mountain, might overwhelm the ability of deterministic processes to constrain community assembly patterns. Our ability to identify dispersal limitation was enabled by the high phylogenetic resolution of the *rpoB* marker that we used, since 16S rRNA analyses provide little ability to resolve patterns of dispersal in *Streptomyces* (Higgins et al., 2021). The existence and impact of dispersal limitation has now been documented for several microbial taxa across a range of ecosystems (Staley and Gosink, 2002; Whitaker et al., 2003; Bell, 2010; Eisenlord et al., 2012; Albright and Martiny, 2017; Bottos et al., 2018; Evans et al., 2019). Hence, it seems likely that microbial dispersal is finite and subject to change over time based on contemporary processes and historical contingencies (Hewitt, 2000; Mennicken et al., 2020; Liao et al., 2021). In the case of *Streptomyces*, we hypothesize that contemporary climate variation is a major determinant of establishment. We also hypothesize that historical variation in climate has contributed significantly to extant patterns of microbial biogeography in North America because rates of dispersal are low relative to rates of climate variation during the Quaternary Period.

## Data Availability Statement

The datasets presented in this study can be found in online repositories. The names of the repository/repositories and accession number(s) can be found below: https://www.ncbi.nlm.nih.gov/, PRJNA790982.

## Author Contributions

JH performed the research, analysis, writing, and editing. DB supervised research and analysis and assisted with writing and editing. Both authors contributed to the article and approved the submitted version.

## Conflict of Interest

The authors declare that the research was conducted in the absence of any commercial or financial relationships that could be construed as a potential conflict of interest.

## Publisher’s Note

All claims expressed in this article are solely those of the authors and do not necessarily represent those of their affiliated organizations, or those of the publisher, the editors and the reviewers. Any product that may be evaluated in this article, or claim that may be made by its manufacturer, is not guaranteed or endorsed by the publisher.

## Supplementary Material

Supplementary Figure 1 Rarefaction curves show that most sites within WM and all WL sites have been sampled to saturation.

Supplementary Figure 2 Elevation and horizontal distance are the best predictors of beta diversity at WM as measured by unweighted UniFrac distance along all measured environmental and spatial gradients.
