# Supplementary material for: Elevational Gradients Impose Dispersal Limitation on Streptomyces
Source: Front Microbiol. 2022 May 3;13:856263. doi: 10.3389/fmicb.2022.856263 (PMC9113539; doi:10.3389/fmicb.2022.856263)
Supplement: Supplementary file 1 [file Table_1.docx]

**Supplementary Table 1**. Beta diversity partitioned into nestedness and species turnover components. Sites that start with C (*e.g.,* C1A1) are in WL, and sites that start with S (*e.g.,* S01_R01) are in WM.

| **Site** | **Nestedness** | **Turnover** |
| --- | --- | --- |
| C1A1 | 0.59992064 | 0.30007937 |
| C1B1 | 0.46111111 | 0.43888889 |
| C1C1 | 0.39971062 | 0.50028938 |
| C1D1 | 0.76131746 | 0.13868254 |
| C1E1 | 0.505 | 0.395 |
| C2A2 | 0.56190476 | 0.33809524 |
| C2B2 | 0.8489011 | 0.0510989 |
| C2C2 | 0.359 | 0.541 |
| C2D2 | 0.73936508 | 0.16063492 |
| C2E2 | 0.71912698 | 0.18087302 |
| S01_R01 | 0.88304136 | 0.11695865 |
| S01_R02 | 0.91056514 | 0.08943486 |
| S01_R03 | 0.91351232 | 0.08648768 |
| S02_R01 | 0.92294111 | 0.07705889 |
| S02_R02 | 0.91701172 | 0.08298829 |
| S02_R03 | 1 | 0 |
| S04_R02 | 0.91844278 | 0.08155722 |
| S04_R03 | 0.89807127 | 0.10192873 |
| S05_R01 | 0.90095909 | 0.09904091 |
| S05_R02 | 0.93573208 | 0.06426792 |
| S06_R01 | 0.86658966 | 0.13341034 |
| S06_R02 | 0.90090972 | 0.09909029 |
| S07_R02 | 0.99033767 | 0.00966233 |
| S08_R02 | 0.88061098 | 0.11938902 |
| S08_R03 | 0.85744562 | 0.14255438 |
| S09_R01 | 0.88054955 | 0.11945045 |
| S09_R02 | 0.88321866 | 0.11678134 |
| S09_R03 | 0.90443657 | 0.0955634 |
