# Supplementary figures and images for: Elevational Gradients Impose Dispersal Limitation on Streptomyces
Source: Front Microbiol. 2022 May 3;13:856263. doi: 10.3389/fmicb.2022.856263 (PMC9113539; doi:10.3389/fmicb.2022.856263)

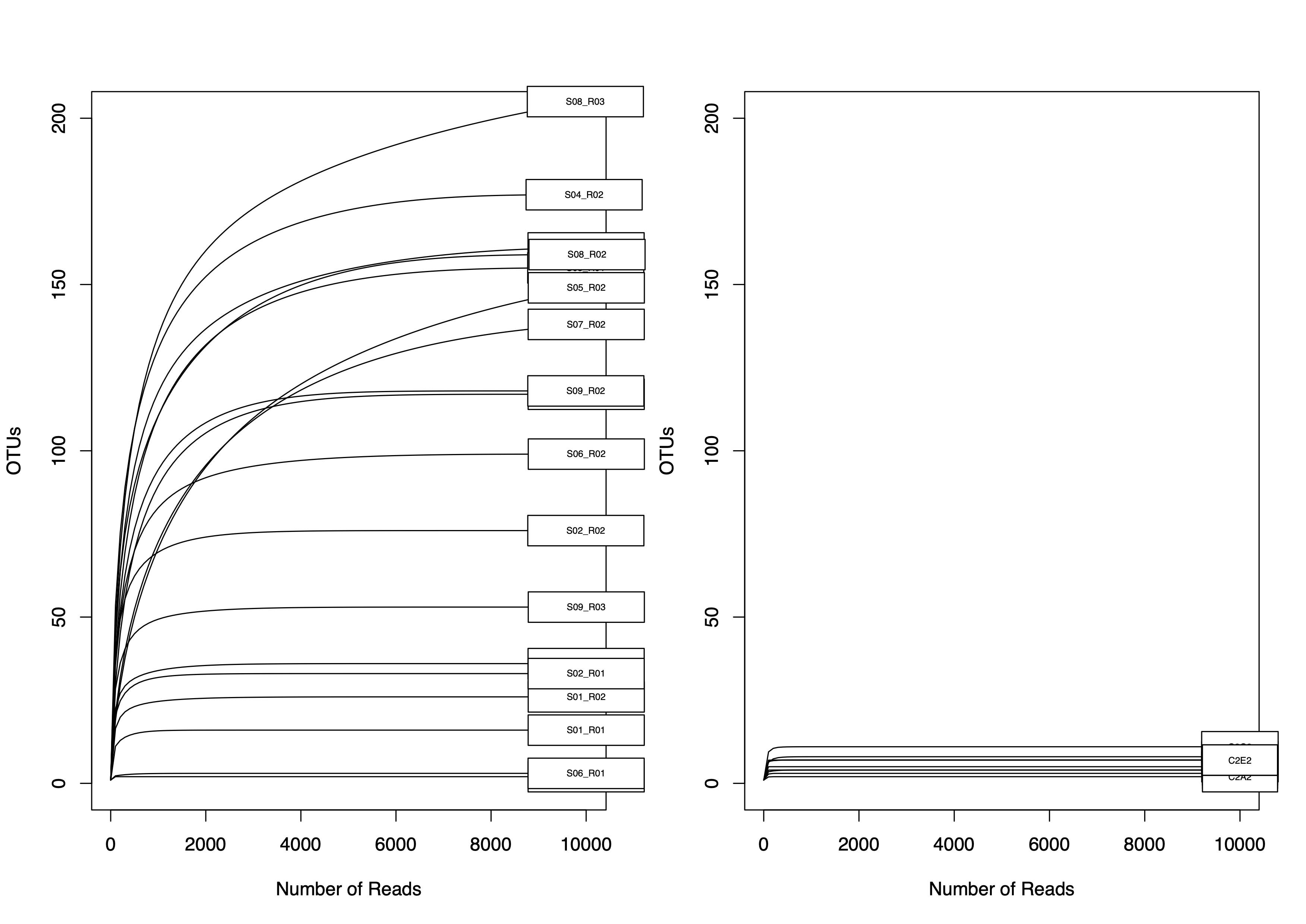

Supplement: Supplementary Figure 1 — Rarefaction curves show that most sites within WM and all WL sites have been sampled to saturation. [file Image_1.JPEG]

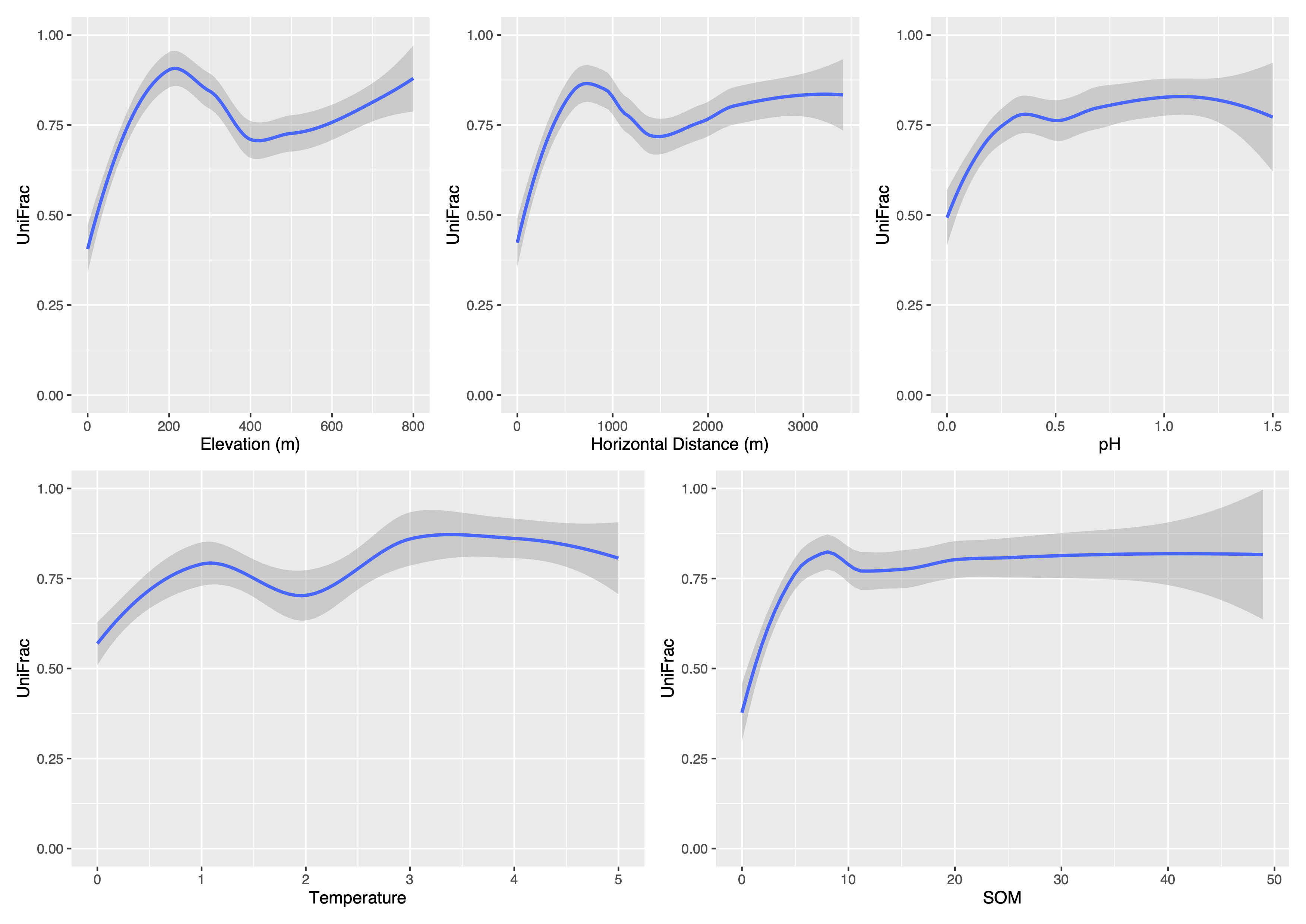

Supplement: Supplementary Figure 2 — Elevation and horizontal distance are the best predictors of beta diversity at WM as measured by unweighted UniFrac distance along all measured environmental and spatial gradients. [file Image_2.JPEG]
